# Addiction to smartphones in Arabs is associated with severe depressive symptoms and insomnia: a cross-sectional study

**DOI:** 10.1007/s44192-024-00123-z

**Published:** 2024-12-19

**Authors:** Omar Gammoh, Salam Shannag, Sereene Al-Jabari, Rama Al-Shawaheen, Saleh Bazi, Ahmed Mohammad Al-Smadi, Albara Mohammad Ali Alomari

**Affiliations:** 1 Department of Clinical Pharmacy and Pharmacy Practice, Yarmouk University, Shafiq Irshidat Str, P O Box 566, Irbid, 21163 Jordan; 2 The University of Jordan, Amman, Jordan; 3 Department of Marketing, Faculty of Business, Yarmouk University, Shafiq Irshidat Str, P O Box 566, Irbid, 21163 Jordan; 4 Refugees Displaced Persons and Forced Migration Studies Centre, Yarmouk University, Shafiq Irshidat Str, P O Box 566, Irbid, 21163 Jordan; 5 Nursing Department, Fakeeh College for Medical Sciences, Jeddah, Saudi Arabia; 6 University of Doha for Science and Technology, Doha, Qatar

## Abstract

Addiction to smartphones is a global issue. Mental health disturbance is an emerging factor implicated in smartphone addiction. Whether depressive symptoms and anxiety are implicated in smartphone addiction in developing countries such as Jordan is a nexus that warrants investigation. The present study investigated whether depressive symptoms and insomnia are correlated with smartphone addiction in a cohort of Jordanian participants. We adopted a cross-sectional design for a convenient sample, smartphone addiction, depressive symptoms, and insomnia were assessed using validated scales. Data analysis from 484 participants revealed that smartphone addiction was reported in 180 (37.1%) of the participants. Symptoms of severe depression and severe insomnia were reported in 174 (35.9%) and 198 (40.9%) of the participants respectively. The multivariable regression analysis showed that severe depressive symptoms and severe insomnia were significantly associated with addiction to smartphones (OR = 1.69, 95% CI = 1.11–2.55, *p* = 0.01) and (OR = 2.19, 95% CI = 1.46–3.29, *p* < 0.001) respectively. In conclusion, poor mental health outcomes are correlated with unhealthy lifestyle habits such as addiction to smartphones, the addressing these alarming mental health symptoms is required to optimize the well-being of the community.

## Introduction

Technological improvements in the twenty-first century extend to widespread use of the internet and artificial intelligence and have become an essential part of our daily activities. Today, mobile phones have an interactive interface, new technological features like cameras, and access to the internet which made most computer duties accessible on a phone anywhere [1].

Smartphones have many advantages; they allow the masses to communicate almost anywhere and anytime, provide means of entertainment; like music and digital games, and increase people's access to high-quality education, navigation, and access to better health with digital doctors [2]. However, smartphones can become a distraction from daily activities, they may pose privacy and security threats, expose teens to bullying, and run the risk of driving and texting [2], Kushlev et al. Associate time spent on mobile phones with general well-being, with addicted users feeling much worse than those who don’t spend time at all [3]. Some literature associates smartphones with eye strain, back pain, and tendonitis alongside insomnia and depressive symptoms [2].

Insomnia is generally defined as discontent in the quality and duration of sleep alongside trouble falling and staying asleep [4]. ISI (insomnia severity score) is used to test for the severity or presence of insomnia. One study found that the shortest sleep duration was a direct result of smartphone and internet use before bedtime [5]. One other study found a positive correlation between the time teens spent on phones and their inability to get 7 h of sleep [6]. A study examined 404 university students in Oman for insomnia, stress, and anxiety in association with smartphone use and concluded that there is a significant association between smartphone use and poor sleep quality [7].

Severe depressive symptoms profoundly disable people from functioning and socializing properly. Defining depressive symptoms is challenging because symptoms can vary, it can present with general symptoms like fatigue and insomnia, and at other times present with anhedonia [8]. Depressive symptoms diagnosis can be made using the Diagnostic and Statistical Manual of Mental Disorders (DSM) or the International Classification of Diseases (ICD) and both test for key symptoms to make a diagnosis. One study analyzed 256 students for problematic phone use and found that screen lock-unlock was associated with increased depressive and anxiety scores [9]. One systematic review analyzed 10 papers studying an association between depressive symptoms and smartphone use and concluded that the severity of depressive symptoms was positively associated with troubled smartphone use regardless of the scale used for analysis [10].

In Jordan as well as several developing countries, the use of smartphones is dramatically growing. Although there are no official data regarding smartphone use, the connections increased by +1.7% between January 2019 and January 2020, equivalent to 81% of the total population [11].

The association between smartphone addiction and mood disorders is still inconclusive. Although some researchers from Jordan related the addiction to smartphones to depression and social isolation [12] (Evon), however, these results have not been replicated in other studies, for example, a cross-sectional study carried out on university students failed to associate smartphone addiction to depression [11]. On the other hand, another study confirmed that depression anxiety, and stress all predicted addiction to smartphones among university students [13].

Despite this emerging evidence, all the previous research coming from Jordan explored this issue only in university students and students. The easiness of use and the attractive applications could be appealing to all age groups to are addicted to smartphones. Therefore, it is essential to pursue an investigation on a sample from the community in Jordan to confirm or to defy the previous findings.

To the best of our knowledge, no previous literature exists that examined severe depressive symptoms and insomnia as potential correlates to smartphone addiction among a representable sample of the Jordanian community. Therefore, the objective of the present study is to examine whether severe depressive symptoms and insomnia are correlated with smartphone addiction in a cohort of Jordanians. We hypothesize that depressive symptoms and insomnia are associated with smartphone addiction among Jordanians.

## Methods

### Study design and settings

This is a cross-sectional study that recruited a cohort of Jordanians using a convenient sampling method. A web-based Google form was developed to collect data. The link directing to the study instrument was shared on different social media platforms such as Facebook and WhatsApp. The study protocol gained approval from XXX University IRB Committee (no. 692). The research team communicated the study's objectives to potential participants. Informed consent was obtained from all individual participants included in the study. Our sample size calculation revealed the need for a minimum of 384 subjects based on a response distribution of 50%, a margin of error of 5%, and a confidence level of 95%.

### Study instrument

A well-structured study tool has been developed for this study. The demographics of the participants were captured, this included sex, age, marital status, employment status, educational level, smoking status, body mass index, prior diagnosis with long-term health conditions, depressive symptoms, and insomnia.

Depressive symptom severity was screened using the Arabic version of the Patient Health Questionnaire (PHQ-9). This validated and reliable (Cronbach alpha = 0.86) tool offers the participants nine questions to answer, these questions are rated against a corresponding score from “0” to “3”, all together, this scale generates a maximum score of 27. A score ≥15 classifies the participant as severely depressed [14, 15].

Insomnia severity was evaluated using the validated and reliable Arabic version of the Insomnia Severity Index (ISI-A) (Cronbach alpha = 0.81). This scale consists of seven elements that assess the severity of insomnia for the past two weeks, the scale generates a score ranging from “0” to “28”, in general, a score ≥15 reflects severe insomnia symptoms [16, 17].

### Outcome variable

The outcome variable of this study was “addiction to smartphones”. This was measured using the Arabic version of the validated and reliable Smartphone Addiction Scale (Cronbach alpha = 0.81) which consists of ten questions rated against Likert scale responses from “1” to “6”, and a cut score ≥39 is used to identify addiction behavior to smartphones [13, 18].

### Data analysis

The frequencies and percentages were used to describe the data presented in the study sample. The associations between the participants’ covariates and the outcome variable were examined preliminarily through Chi-square analysis (Table 1), subsequently, factors showing *p* < 0.10 suggested their potential independent significant association with the dependent variable as in previous studies [15, 19], were used to feed the multivariable binary logistic regression model for smartphone addiction as the dependent variable, the model was build using the backward stepwise method. The model first included “age”, “marital status”, “Are you a student?”, “depressive symptoms”, and “insomnia” as potential factors. Finally, only the significant correlates (*p* < 0.05) were kept in the final model as in Table 2. Significance was set at *p* < 0.05 and confidence intervals at 95%. Data were analyzed using Spss.

**Table 1:** Association between the participants’ factors and addiction to smartphones using chi-square test (n = 484)

Factor	Category	Non addicted to smartphone n (%)	Addicted to smartphone n (%)	Chi-square score	*p*-value
Sex	Male	67 (22)	38 (21.2)	0.03	0.90
	Female	238 (78)	141 (78.8)		
Age	18–25 years	194 (63.6)	129 (71.7)	3.31	0.04
	25 and above	111 (36.4)	51 (28.3)		
Marital status	Single	222 (73)	143 (79.9)	2.87	0.06
	Married	82 (27)	36 (20.1)		
BMI	<25	184 (60.3)	108 (60)	0.005	0.51
	25 and above	121 (39.7)	72 (40)		
Smoking	Non-smoker	223 (73.1)	123 (68.7)	1.07	0.17
	Smoker	82 (26.9)	56 (31.3)		
Educational level	University	275 (90.2)	161 (89.9)	0.006	0.53
	Highschool	30 (9.8)	18 (10.1)		
Are you a student?	No	147 (48.4)	72 (40.2)	3.00	0.05
	Yes	157 (51.6)	107 (59.8)		
Diagnosed with chronic diseases?	No	271 (88.9)	162 (90.5)	0.32	0.64
	Yes	34 (11.1)	17 (9.5)		
Severe depressive symptoms	No	217 (71.1)	94 (52.2)	7.62	0.04
	Yes	88 (28.9)	86 (47.8)		
Severe insomnia	No	207 (67.9)	80 (44.4)	25.7	<0.001
	Yes	98 (32.1)	100 (55.6)		

*BMI* body mass index

**Table 2:** Multivariable binary logistic regression analysis for “smartphone addiction” as the dependent variable showing the significant correlates

Independent variable	B	OR	95% CI	*p*-value
Severe depressive symptoms	0.52	1.69	1.11–2.55	0.01
Severe insomnia	0.78	2.19	1.46–3.29	<0.001

Depressive symptoms measured using the PHQ-9 scale with a score of 15 and higher for severe depressive symptoms, Insomnia measured using the ISI-A scale with a score of 15 and higher for severe insomnia, Smartphone addiction (the dependent variable) measured using the smartphone addiction scale

## Results

### Study sample characteristics

A total of 484 participants were included in the analysis, 379 (78.8%) were females, 323 (66.7%) were aged between 18 and 25 years old, 365 (75.4%) were single, 292 (60.3%) reported a BMI < 25, 264 (54.5%) were university students. In addition, our results revealed that smartphone addiction was reported in 180 (37.1%) of the participants, severe depressive symptoms were reported in 174 (35.9%) participants, and severe insomnia in 198 (40.9%) participants. Please refer to Table 1.

### Correlates of smartphone addiction

According to the multivariable binary logistic regression results as shown in Table 2 where addiction to smartphones was the dependent variable, it was demonstrated that participants reporting severe depressive symptoms i.e. PHQ-9 score ≥15 were at significantly higher odds (OR = 1.96, 95% CI = 1.11–2.55, *p* = 0.01) for smartphone addiction, also, participants reporting severe insomnia symptoms according to the ISI-A scale scoring score ≥15 were at a significantly higher odds (OR = 2.19, 95%CI = 1.46–3.29, *p* < 0.001) for smartphone addiction. Please refer to Table 2.

## Discussion

Addiction to smartphones is a global issue that warrants investigation. The current investigation estimated the prevalence of addiction to smartphones and identified depressive symptoms and insomnia as correlates.

The prevalence of smartphone addiction was reported to be 36.2 and 37.2% in Jordanian males and females respectively, with no significant difference between both genders. These results are compatible with several studies conducted all around the world showing that gender doesn’t have any impact on the prevalence of addiction, but rather on factors influencing it and its patterns of use which needs to be assessed in further studies [20–22] studies.

In terms of age, participants aged <25 years were significantly more addicted to smartphones than older participants. Previous studies showed that addiction is age dependent and there is a significant decline in problematic smartphone usage and growing up [23–25] This could be attributed to several factors such as using social media more frequently, the fear of missing out (FOMO) besides using smartphones in education [24].

This study showed that about 60% of smartphone-addicted participants were students; this could be explained by the rapidly accelerated use of technology in education, and that the use of smartphones provides instant access to a vast array of academic resources, e-books and articles along with educational apps used in distance learning, especially after the COVID-19 pandemic [26, 27].

In this study, smartphone addiction was not associated with the level of education contrary to previous studies whereas people with higher education were not addicted to smartphone use compared to other people. Most studies assessing the association between educational level and smartphone addiction were conducted in the past decades where people with high education tended to use phones for making calls, sending emails, and reading news only. The evolution of smartphone apps including social media apps and video games could explain the lack of association between the educational level and smartphone addiction.

As reported by this study, marital status doesn’t affect addiction to smartphones which is opposed to what has been found in previous studies about the Asian and Middle Eastern populations [28, 29]. These controversial results might be explained by the difference in sample size and that most studies included participants with specific professions or age groups while this study included general participants of different age groups, educational levels, and professions.

Data analysis from a representative sample from the Jordanian community (n = 484) participants revealed that smartphone addiction was reported by 180 (37.1%) of the participants, the multivariable regression analysis showed that severe depressive symptoms were significantly associated with smartphone addiction (OR = 1.69, 95% CI = 1.11–2.55, *p* = 0.01). Consistent with previous studies conducted on university students in Jordan and people living in the Middle East region and other countries all over the world, the severity of depressive symptoms is increasing significantly with the level of smartphone addiction [30–32] addiction. A study conducted in Turkey showed that those who use social media apps 3–4 h a day show more depressive features than those who use them 1–2 h [33]. Smartphone addicts may get depressive symptoms due to a lack of interaction with other people in real life or due to comparing their lives with people on social media and the feeling of worthlessness and inferiority.

Also, severe insomnia in this study was significantly associated with smartphone addiction (OR = 2.19, 95% CI = 1.46–3.29, *p* < 0.001). In addition to its association with smartphone addiction, early and middle insomnia is used now as a predictor of smartphone addiction as shown by the Chen study [34]. Smartphone addiction causes poorer and shorter nighttime sleep, excessive daytime sleep, and late bedtime [7, 35–37]. The proposed reasons that cause insomnia in smartphone addicts are postponing sleep time, mental stimulation, or exposure to phone light.

According to the literature, the relationship between mental distress and addiction to smartphones seems complex and bi-directional, for example, pre-existing depressive symptoms and anxiety were exacerbated by smartphone addiction, and vice versa, individuals with increased smartphone use showed elevated levels of anxiety when they were away from their phones. Also, individuals with social anxiety who utilize their smartphones to avoid real-life social interactions might prolong this anxiety by falling into this cycle of smartphone use [38].

The present investigation contributes to the existing literature by underscoring the importance of tackling mental health issues in the community, especially in developing countries such as Jordan. The sample size of the current study, the validated scales, and the rigorous data analysis are all strengths, however, there are some limitations. For example, the cross-sectional design precluded the assessment of mental health scores in a longitudinal fashion, also, the criteria used in identifying addiction to smartphones could be employed using other scales [39], in addition, the self-rated scales could yield overrated prevalences.

## Conclusion

This study highlights the association between smartphone addiction and depressive symptoms and insomnia along with their correlates. The severity of depressive symptoms and insomnia increases significantly with the severity of smartphone addiction. Hence most smartphone addicts are young males and females, and since addiction affects their mental health and ultimately their school performance and work productivity, it is necessary to increase the awareness of Jordanian people about this issue and make decisions that might help with decreasing time spent on smartphones. Moreover, further research should be conducted to test the patterns of smartphone use and their short- and long-term effects on other mental health problems such as decreased concentration, anxiety, and memory impairment especially in children and teenagers to improve the well-being of the community.

## Data Availability

The data supporting this study's findings are not openly available due to reasons of sensitivity and are available from the corresponding author upon reasonable request.
